# Oxygen stress reduces zoospore survival of *Phytophthora* species in a simulated aquatic system

**DOI:** 10.1186/1471-2180-14-124

**Published:** 2014-05-13

**Authors:** Ping Kong, Chuanxue Hong

**Affiliations:** 1 Virginia Tech, Hampton Roads Agricultural Research and Extension Center, Virginia Beach, VA 23455, USA

**Keywords:** *Phytophthora* species, Aquatic ecology, Zoospore survival, Dissolved oxygen

## Abstract

**Background:**

The genus *Phytophthora* includes a group of agriculturally important pathogens and they are commonly regarded as water molds. They produce motile zoospores that can move via water currents and on their own locomotion in aquatic environments. However, zoosporic response to dissolved oxygen, an important water quality parameter, is not known. Like other water quality parameters, dissolved oxygen concentration in irrigation reservoirs fluctuates dramatically over time. The aim of this study was to determine whether and how zoospore survival may be affected by elevated and low concentrations of dissolved oxygen in water to better understand the aquatic biology of these pathogens in irrigation reservoirs.

**Results:**

Zoospores of *P. megasperma*, *P. nicotianae*, *P. pini* and *P. tropicalis* were assessed for survival in 10% Hoagland’s solution at a range of dissolved concentrations from 0.9 to 20.1 mg L^-1^ for up to seven exposure times from 0 to 72 h. Zoospore survival was measured by resultant colony counts per ml. Zoospores of these species survived the best in control Hoagland’s solution at dissolved oxygen concentrations of 5.3 to 5.6 mg L^-1^. Zoospore survival rates decreased with increasing and decreasing concentration of dissolved oxygen, depending upon *Phytophthora* species and exposure time. Overall, *P. megasperma* and *P. pini* are less sensitive than *P. nicotianae* and *P. tropicalis* to hyperoxia and hypoxia conditions.

**Conclusion:**

Zoospores in the control solution declined over time and this natural decline process was enhanced under hyperoxia and hypoxia conditions. These findings suggest that dramatic fluctuations of dissolved oxygen in irrigation reservoirs contribute to the population decline of *Phytophthora* species along the water path in the same reservoirs. These findings advanced our understanding of the aquatic ecology of these pathogens in irrigation reservoirs. They also provided a basis for pathogen risk mitigation by prolonging the turnover time of runoff water in recycling irrigation systems via better system designs.

## Background

*Phytophthora* species, a group of fungal-like destructive plant pathogens, are known as water molds [1-4]. They produce motile zoospores that can spread through irrigation systems from runoff water retention basins at ornamental crop production facilities and cause severe plant diseases and crop losses. Over 40 species of *Phytophthora* have been recovered from irrigation systems and natural waterways [5].

Zoospores generally are short-live and their survival is subject to environmental stresses. Majority of zoospores survive for less than 24 h [6-8]. Zoospore survival of individual species in aquatic environments depends upon water pH [7,9], electrical conductivity (EC) [6], and CO_2_[10,11].

Dissolved oxygen is another important water quality parameter. Dissolved oxygen concentration in agricultural reservoirs varies among water sources and fluctuates seasonally as well as diurnally within the same sources due to activities of phytoplankton, change of temperature and atmosphere pressure [12]. Dissolved oxygen concentration in lakes, streams, and ponds that receive runoff from nurseries was 9.0, 7.0 and 12.0 mg L^-1^, respectively [13]. Dissolved oxygen concentrations in runoff water containment basin that was also an irrigation reservoir varied from 0.3 to 26.5 mg L^-1^ over time [13]. These oxygen concentrations are much lower than the atmospheric oxygen level of 21% or 276 mg L^-1^ based on the air density of 1.2 g m^-3^ with 23.2% of oxygen at the sea level (http://www.en.wikipedia.org/wiki/Atmosphere_of_Earth).

Dissolved oxygen is known to affect the survival of fish and other aquatic organisms including algae [14]. Whether and how dissolved oxygen may affect zoospore survival of *Phytophthora* species in irrigation reservoirs is not known. Previous studies in relation to oxygen have focused primarily on other propagules in terrestrial rather than zoospores in aquatic environments. Species of *Phytophthora* grew well in oxygen concentrations from 0.04% to 21% (or 0.5–276 mg L^-1^) in soil or on agar media [15,16]. Mycelia can grow under a wide range of oxygen conditions as long as its concentration was below 1.6% (or 21 mg L^-1^) [15,17]. However, *Phytophthora* species produce sporangia in water films under a narrow range of dissolved oxygen concentrations. For instance, sporangium production was prolific at an oxygen level of 5% (or ≥ 65 mg L^-1^) but production nil to few at 1% (or 13 mg L^-1^) [18]. Few oospores were produced at atmospheric oxygen levels of 276 mg L^-1^ or higher while numerous were produced at much lower levels at 13 and 65 mg L^-1^[16,17,19]. Disease development delayed in plants inoculated with *P. cinnamomi* at an oxygen range of 0.9–2.3 mg L^-1^ in aeroponic and hydroponic systems [20,21]. These studies demonstrate that different propagules may require different levels of oxygen for production, growth and survival.

Here, we report the effects of elevated and low concentrations of dissolved oxygen in a simulated aquatic system on zoospore survival for several *Phytophthora* species. The aim of this study was to develop a better understanding of aquatic ecology of *Phytophthora* species, establishing a base for devising sustainable mitigation strategies for these pathogens in irrigation water.

## Methods

### Base medium, dissolved oxygen treatment systems

The base medium used for all the experiments in this study was 10% Hoagland’s solution. The full strength solution was prepared with Hoagland’s basal salt mixture (MP Bio, Solon, OH, USA) and adjusted with NaOH to have a final pH of 7.0. To maintain a stable pH, the stock solution was buffered with 1 mM MES hydrate (Sigma, St. Louis, MO USA) and stored at 4°C until use. The stock solution was freshly diluted with dH_2_O at 1:10. The diluted solution was then placed in 500-ml glass bottles leaving no or little room for air. Bottle filling was done 18–20 h ahead of experiment to allow temperature equilibrium. As measured with EcoSense® DO 200 meter (YSI Inc, South Burlington, VT, USA), dissolved oxygen concentration in the control solution (CK) as static 10% Hoagland’s solution at 23°C was 5.3 to 5.6 mg L^*-*1^.

### Potential side effect of nitrogen as replacement gas on zoospore survival

Although nitrogen does not react with water it dissolves in water at 20 mg L^-1^at 20C (http://www.lenntech.com/periodic/water/nitrogen/nitrogen-and-water.htm). To determine whether dissolved N_2_ in the solution from bubbling pure N_2_ directly affects zoospore survival, assays were performed with four selected *Phytophthora* species. Three treatments were included: (i) CK–the control Hoagland’s solution, (ii) N_2_–the same solution bubbled with pure N_2_ for 10 min to reduce dissolved oxygen concentration to 0.9 mg L^-1^, and (iii) dN_2_–the bubbled solution with N_2_ for 10 min was poured into open containers allowing to restore dissolved oxygen concentration to 5.3 mg L^-1^ over a 48-h period. The details of species and isolates as well as the zoospore survival assay protocol are described below. For simplicity, only data from *P. tropicalis* are presented.

### Elevation and reduction of dissolved oxygen concentration in the base medium

Dissolved oxygen elevation and reduction was achieved by bubbling pure oxygen (O_2_) or nitrogen (N_2_) into 10% Hoagland’s solution in the bottles. For dissolved oxygen concentration elevation, oxygen was bubbled at 0.5 L min^-1^ for 0, 15, 30, 45, 60, 75, 90, 120 or 150 seconds. Dissolved oxygen concentrations were measured immediately after bubbling. This experiment was repeated three times. The dissolved oxygen concentration in the solution after bubbling 90 seconds were out of range of the DO 200 meter which can measure up to 18 mg L^-1^. Data from repeating experiments were pooled after homogeneity test. Prior to the further analysis, bubbling time was divided into 15-second segments and assigned numerical values with 1 for the first (0-15 seconds), 2 for the second (16-30 seconds), and 5 for the fifth (61-75 seconds). Correspondingly, dissolved oxygen elevation was computed for individual 15-second time segments with 3.2, 2.4, 2.2, 1.8, and 1.5 mg L^-1^ for the first, second, third, fourth and fifth (Table 1). The speed of dissolved oxygen concentration elevation was then related to these 15-second time segments using Proc GLM (SAS Institute, Cary, North Carolina, USA). A resultant model was used to make elevated concentrations of dissolved oxygen as desired in the zoospore survival assays. To track the dynamics of dissolved oxygen concentration in the solutions, additional measurements were taken at 2, 4, 8 and 24 h following oxygen bubbling. All bottles were sealed with parafilm then capped tightly after bubbling and each measurement.

**Table 1:** **Dissolved oxygen (DO) levels in 10% Hoagland’s solution generated by oxygen (O**_**2**_**) or nitrogen (N**_**2**_**) bubbling**

**O**_**2 **_**bubbling at 0.5 L min**^**-1**^						**N**_**2 **_**bubbling at 0.4 L min**^**-1**^		
**Time (Sec)**	**Assigned time segment value (x)**	**Measured DO (mg L**^**-1**^**)**^**y**^	**SD**	**Predicted DO increase within time segment (y)**^**Z**^	**Predicted total DO in solution**	**Time (Min)**	**Measured DO (mg L**^**-1**^**)**	**SD**
0	0	5.6	0.2	-	5.6	0	5.3	0.1
15	1	8.8	0.0	3.2	8.8	2	2.0	0.0
30	2	11.2	0.2	2.5	11.3	5	1.2	0.0
45	3	13.4	0.3	2.1	13.4	10	0.9	0.1
60	4	15.2	0.2	1.8	15.4	20	0.9	0.0
75	5	16.7	0.2	1.6	16.7	30	1.0	0.1
90	6	Out of range	ND	1.4	18.1			
120	8	Out of range	ND	1.1	19.2			
150	10	Out of range	ND	0.9	20.1			

^y^These numbers are meter readings and the meter cannot measure dissolved oxygen above 18.0 mg L^-1^.

^Z^These values are calculated based on a regression model: y = 3.2 - ln (x), as generated from the SAS analysis.

For dissolved oxygen reduction, pure nitrogen gas was bubbled into the Hoagland’s solution in the bottles at 0.4 L min^-1^ for 2, 5, 10, 20, or 30 min. Dissolved oxygen concentrations were measured immediately after bubbling subsequently selected for the zoospore survival studies. Similarly, the dynamics of dissolved oxygen concentration in the solutions was tracked following the N_2_ bubbling.

### *Phytophthora* species and zoospore suspension preparation

Irrigation water isolates of four *Phytophthora* species: *P. megasperma* (isolate 42D2), *P. nicotianae* (45H1), *P. pini* (previously, *P. citricola*, 43H1) and *P. tropicalis* (7G9) were used in this study [7]. These species had differential responses to pH stress [22].

Cultures were maintained and zoospore suspensions were prepared as described previously [7]. Briefly, zoospore suspension was prepared with agar plugs from one-week-old cultures. The plugs were grown in 10% clarified V8 juice broth at room temperature for 7 days for *P. nicotianae* and *P. tropicalis*, and 3 days for *P. megasperma* and *P. pini*. After the media were removed, the cultures were then rinsed with sterile distilled water (SDW), drained and exposed to fluorescent light for 24 - 48 h for *P. nicotianae* and *P. tropicalis*, 8 h for *P. megasperma*. For *P. pini*, the cultures were flooded with SDW again then incubated under lights for 8 h to facilitate sporangium production. After the light exposure, water was drained then plates were refilled with chilled sterile soil water extract to trigger zoospore release. Zoospore yields reached > 10^4^ mL^-1^ after 30 min for *P. nicotianae* and *P. tropicalis*, and after overnight for *P. megasperma* and *P. pini*. Zoospore suspensions were filtered through a layer of sterile miracloth to remove cultural plugs and mycelia. Zoospore concentrations were determined with a haemocytometer.

### Zoospore survival assays

Three sets of zoospore survival assays were performed to determine the impacts of (i) potential side effect of nitrogen as a replacement gas for oxygen in the Hoagland’s solutions, (ii) elevated and (iii) low concentrations of dissolved oxygen in comparison with the regular concentration in the control solutions that were not bubbled with any gas (O_2_ or N_2_). The elevated concentrations of dissolved oxygen tested were 11.3, 15.2, 18.1, 19.2, 20.1 mg L^-1^, and the normal concentration of 5.6 mg L^-1^ (control) along with reduced concentrations of dissolved oxygen at 2.0, 1.2, and 0.9 mg L^-1^. The dissolved oxygen treatments were made as described above.

A certain volume of fresh zoospore suspension was added to each bottle to make a final concentration of 50 zoospores mL^-1^ without altering the dissolved oxygen concentration in the Hoagland’s solutions. Bottles were gently inverted twice then two or three 1-mL aliquots were taken out from each bottle within 10 min. Each aliquot was spread onto a 90-mm plate with PARP-V8 agar [23]. Additional samples were taken at 2, 4, 8, and 24 h in the elevated dissolved oxygen assays. Two more samples were taken for the reduced dissolved oxygen assays at 48 or 72 h, respectively. The plates were placed at room temperature for 2 to 3 days. Emerging colonies in each plate were counted and the colony counts were used to measure zoospore survival in the Hoagland’s solutions at various concentrations of dissolved oxygen for different exposure times. Each experiment included three replicate bottles and was repeated at least three times.

### Statistical analyses of zoospore survival assay data

Data of zoospore survival rates as measured by resultant colony counts from repeating assays were examined for homogeneity then analyzed separately with Proc ANOVA. Mean survival rates of three replicates from 6 or 9 plates were separated by the least significant difference (LSD) at *P* = 0.05. Linear regression analyses were performed to determine whether and how the elevated concentrations of dissolved oxygen may affect the colony counts by *Phytophthora* species and exposure time. Similar analyses also were conducted to determine whether and how the level of dissolved oxygen reduction in the Hoagland’s solutions from its normal concentration (5.3 mg L^-1^) may influence the colony counts of four *Phytophthora* species at different exposure times.

## Results and discussion

### Effect of dissolved nitrogen on zoospore survival

In preliminary studies using hydrazine hydrate and CO_2_ to manipulate dissolved oxygen concentration in Hoagland’s solution, we found that both chemicals themselves significantly reduced zoospore survival [10,22]. Nitrogen was used as replacement gas for oxygen in previous investigations into the mycelial growth of *Aphanomyces euteiches*[24], *Phytophthora cactorum*[15], *Pythium ultimum*[25], and spore germination of *Phytophthora citrophthora*, *Phytophthora nicotianae*, and *Thielaviopsis basicola* in liquid medium [17,26]. Nitrogen also was used in hydroponic systems to investigate root infection of avocado (*Persea americana*), shortleaf pine (*Pinus echinata*) and loblolly pine (*Pinus taeda*) by *Phytophthora cinnamomi*[21,27,28]. However, none of these studies evaluated the potential impact of high concentration of nitrogen itself. Thus, the first assay performed was to determine whether nitrogen itself impacts zoospore survival.

**Figure 1:**
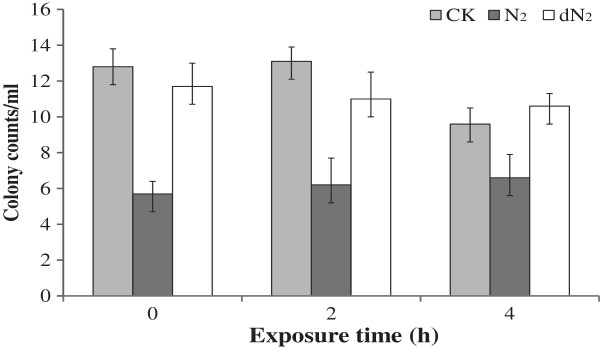
**Impact of dissolved N**_**2 **_**and oxygen on zoospore survival of *Phytophthora tropicalis*.** CK, 10% Hoagland’s solution (pH 7) at dissolved oxygen (DO) of 5.3 mg L^-1^ without N_2_ bubbling; N_2_, same solution bubbled with N_2_ for 10 min to reduced DO to 0.9 mg L^-1^; dN_2_, same solution bubbled with N_2_ for 10 min then aerated until DO returned to 5.3 mg L^-1^; Each column is a mean of the three replicates, topped with standard deviations of the mean.

Hoagland’s solution at 10% strength was used as base medium and four species of *Phytophthora* were included in this assay. Zoospore survival was compared among three solutions: (i) control solutions (CK) as a static 10% Hoagland’s solution with dissolved oxygen at 5.6 mg L^-1^, (ii) bubbled with nitrogen (N_2_) to reduce dissolved oxygen concentration to 0.9 mg L^-1^, and (iii) degassed after nitrogen bubbling (dN_2_) with a final concentration of dissolved oxygen similar to that in the control solution. No difference in colony counts was observed between the control and degassed solutions (dN_2_) regardless of exposure time as illustrated by *P. tropicalis* (Figure 1). As expected, more colony counts were consistently resulted from the degassed solutions (dN_2_) than those not degassed (N_2_) solutions (Figure 1). These results indicate that dissolved nitrogen in the Hoagland’s solution had no effect on the zoospore survival. Similar results were obtained for the other three species evaluated in this study. These results implicate nitrogen had no impact on spore germination, mycelial growth, and root infection of avocado and pines in those previous studies [15,17,21,24,25,27,28] and it is a good replacement gas for the subsequent assays in this study.

### Elevation and reduction of dissolved oxygen concentration with gas bubbling

The second assays conducted were to establish the relationship between dissolved oxygen concentration and gas bubbling time and to understand the post-bubbling dynamics of dissolved oxygen concentration in the solutions. Dissolved oxygen concentrations in the 10% Hoagland’s solution increased with increasing oxygen bubbling time (Table 1). But the speed of dissolved oxygen elevation in the solution decreased at every additional 15-second segment of bubbling time. This relationship was best fitted (R = 0.9842) as:

$$y=3.2–\ln\;\left(x\right)$$

in which y is the speed of dissolved oxygen elevation (mg L^-1^) per 15 seconds; x is the number of 15-second segments (x > 0).

The concentrations of dissolved oxygen in the solutions after being bubbled with oxygen for 90, 120 and 150 seconds were estimated to be 18.1, 19.2, and 20.1 mg L^-1^, respectively (Table 1).

Dissolved oxygen concentrations decreased with increasing nitrogen bubbling time up to 10 minutes (Table 1). Extended nitrogen bubbling for 20 and 30 min did not further decrease the dissolved oxygen concentration in the Hoagland’s solutions (Table 1). Thus, these 20 and 30 min treatments were excluded from the subsequent studies.

**Figure 2:**
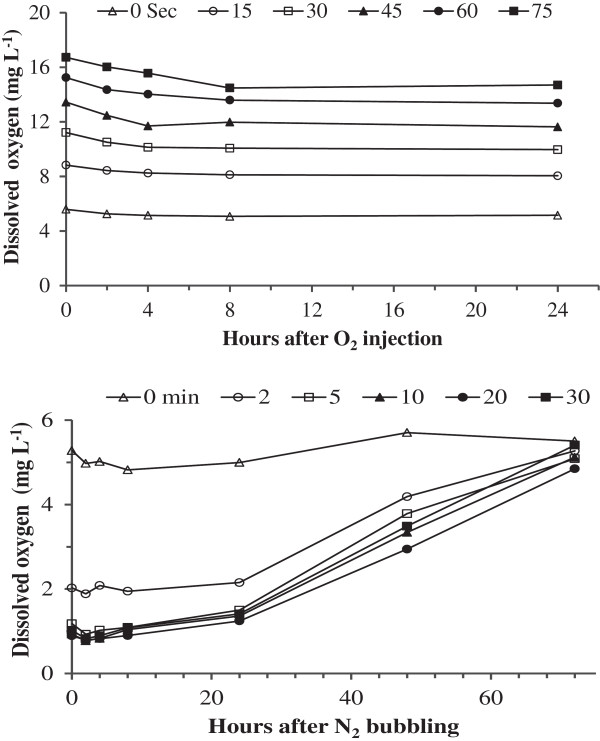
**Dynamics of dissolved oxygen levels in 10% Hoagland’s solution following O**_**2 **_**(top) and N**_**2 **_**(bottom) bubbling.**

There was little change in the dissolved oxygen concentration within the 24 h of oxygen and nitrogen bubbling (Figure 2). However, dissolved oxygen concentration in the Hoagland’s solutions was gradually restored to its original concentration of 5.3 to 5.6 mg L^-1^ within 72 hours of bubbling regardless of gas treatment (O_2_ or N_2_).

### Effect of elevated concentrations of dissolved oxygen on zoospore survival

Among the four species assessed in this study, only zoospores of *P. megasperma* in the control bottles at dissolved oxygen concentration of 5.6 mg L^-1^ consistently declined with increasing exposure time as reflected in the intercept of the linear models (Table 2). The greatest colony count of this species was observed at 10-min and 2-h exposures and the least at 24-h exposure. It is not known at this time why the greatest colony counts of *P. nicotianae, P. pini* and *P. tropicalis* occurred at 2- or 4-h instead of 10-min exposures.

**Table 2:** **Linear regression analyses of colony counts (y) and elevated concentrations of dissolved oxygen in the Hoagland’s solutions (x) after being bubbled with pure oxygen by *Phytophthora *** **species and exposure time**^**z**^

**Species**	**Exposure (h)**	**Intercept ( *a *)**	**Slope ( *b *)**	***P***
*P. megasperma*	0 (10 min)	24.1	-0.4	< 0.0001
	2	22.0	-0.3	0.0010
	4	15.3	-0.2	0.0324
	8	11.9	-0.2	0.4980
	24	9.5	0.1	0.1902
*P. nicotianae*	0	2.8	0.2	0.0032
	2	23.5	-0.4	0.0011
	4	33.0	-0.7	0.0001
	8	22.5	-0.2	0.0377
	24	7.0	0.2	0.0202
*P. pini*	0	7.6	0.3	0.0032
	2	42.3	-0.9	0.0033
	4	43.1	-1.4	< 0.0001
	8	21.2	-0.3	0.0175
	24	17.7	-0.4	0.0006
*P. tropicalis*	0	13.3	-0.2	0.0794
	2	21.2	-0.4	0.0025
	4	22.0	-0.6	0.0004
	8	17.7	-0.3	0.0098
	24	10.2	-0.4	< 0.0001

^z^Linear model: y = *a* + *b*x, in which x ≥ 5.6 mg L^-1^.

As indicated by the slope of linear models, zoospore survival of all four species were negatively impacted by elevated concentrations of dissolved oxygen for most exposure times (Table 2). For instance, the colony counts of *P. megasperma* decreased with increasing dissolved oxygen concentration at 10-min (*P* < 0.0001), 2-h (*P* = 0.0010) and 4-h exposures (*P* = 0.0324). The colony counts of the other three species decreased with increasing dissolved oxygen concentration at all exposure times with a few exceptions. As indicated by the slope of linear models, the greatest rate of decrease in colony counts occurred at 4-h exposure with 0.7 colony per unit of dissolved oxygen increase for *P. nicotianae* (*P* = 0.0001), 1.4 for *P. pini* (*P* < 0.0001), and 0.6 for *P. tropicalis* (*P* = 0.0004), respectively. The only exceptions were observed in *P. megasperma* at 24 h, *P. nicotianae* at 10 min and 24 h, as well as *P. pini* at 10 min.

These results indicated that zoospore survival in runoff water containment basins is subjected to fluctuations of dissolved oxygen concentration in particular of hyperoxia conditions although there are slightly differences among the four species assessed in this study. *P. megasperma* was least affected by elevated concentrations of dissolved oxygen as was by a range of pH in a previous study [7]. Differences in oxygen response were previously observed among oomycetes and fungi. By their oxygen response, these fungi and oomycetes can be grouped into three categories. First, mycelial growth is directly proportional to atmospheric oxygen level with the optimum at 21.0%. This pattern is exemplified by *P. nicotianae* (syn. *P. parasitica*)*, P. citrophthora* and *T. basicola*[17] and *P. cactorum*[15]. Second, mycelial growth has a clear optimal oxygen level typically well below 21.0%, which distinguishes this group from those of the first pattern. Examples of this group included *A. euteiches* that had optimal growth at 5.0% [24]. Third, mycelial growth increases with increasing atmospheric oxygen only to a concentration, above which results in no further growth benefits. This pattern is illustrated by *P. ultimum*, of which mycelial growth was reduced at oxygen concentration of 1.3% but was the same for all oxygen levels from 4.0% to 21.0% [25].

It is unclear how the elevated concentrations of dissolved oxygen affected zoospore survival of different species. In this study we did observe that zoospores of *P. nicotianae*, *P. pini* and *P. tropicalis* remained motile for more than 2 h after their release from sporangia while the most zoospores of *P. megasperma* had already encysted before they were added to the 500-ml volume at the various dissolved oxygen concentrations. It is reasonable to assume that motile zoospores are more vulnerable to environmental stresses including elevated concentrations of dissolved oxygen or hyperoxia than those encycled ones with cell wall.

It is worth of noting that zoospores of *P. nicotianae* died instantly in a 9.5-L fish tank being bubbled with oxygen at 0.5 L min^-1^ for 20 min under a separate experiment [22]. The dissolved oxygen concentration in this fish tank was estimated to be over 27.3 mg L^-1^ according to the formula developed above. It also was previously reported that hyperoxia suppressed fungi and bacteria [29,30].

Artificial oxygenation of irrigation water for pathogen mitigation may not be economically feasible. However, dissolved oxygen concentration in irrigation reservoirs can naturally rise up to 26.5 mg L^-1^ due to phytosynthetic activities [13]. Zoospores are the principal, if not sole, dispersal and infective propagules of *Phytophthora* and *Pythium* species in recycling irrigation systems [31-35]. Thus, the results of present study, along with those of previous studies [15,17,21,24,25,27,28], help understand the dynamics of these pathogens in irrigation reservoirs under hyperoxia conditions [36,37].

### Effect of low concentrations of dissolved oxygen on zoospore survival

As in the dissolved oxygen elevation assays, the greatest colony counts in the control bottles occurred at 10-min exposure for *P. megasperma* and at 2- or 4-h exposure for the other three species (Table 3).

**Table 3:** **Linear regression analyses of colony counts (y) and levels (x) of dissolved oxygen reduction from that in the control Hoagland’s solution by *Phytophthora *** **species and exposure time**^**z**^

**Species**	**Exposure (h)**	**Intercept ( *a *)**	**Slope ( *b *)**	***P***
*P. megasperma*	0 (10 min)	18.2	-1.0	0.0936
	2	11.3	-0.2	0.6267
	4	9.9	-0.8	0.0104
	8	7.4	-0.3	0.2903
	24	8.4	-0.7	0.0292
	48	7.6	-0.9	0.0015
	72	4.5	-0.3	0.0724
*P. nicotianae*	0	7.8	0.8	0.1067
	2	25.0	-1.2	0.0548
	4	28.5	-2.6	0.0008
	8	12.3	-0.4	0.4421
	24	5.1	-0.2	0.4100
	48	3.6	0.0	0.8670
	72	2.2	0.1	0.3973
*P. pini*	0	9.1	0.4	0.2462
	2	32.6	-0.3	0.6893
	4	37.2	-2.1	0.0002
	8	20.8	-1.3	< 0.0001
	24	14.4	-0.8	0.0034
	48	7.4	-0.3	0.2382
	72	8.3	-0.5	0.0313
*P. tropicalis*	0	27.8	-1.8	0.0156
	2	31.4	-1.3	0.0749
	4	29.7	-0.3	0.6712
	8	22.5	-0.1	0.8042
	24	7.8	-0.3	0.1730
	48	0.7	0.4	0.0008
	72	0.4	0.2	0.0079

^z^Linear model: y = *a* + *b*x, in which x = 5.3 - meter readings of dissolved oxygen in the Hoagland’s solutions after being bubbled with pure nitrogen, so 0 ≤ x ≤ 5.3 mg L^-1^.

Zoospore survival of the four species assessed in this study also was negatively impacted by low concentrations of dissolved oxygen in two distinct patterns (Table 3). One pattern is represented by *P. megasperma* and *P. pini*. The impact on these two species generally occurred at 4-h or longer exposures at which their colony counts decreased with increasing level of dissolved oxygen reduction from the normal concentration of 5.3 mg L^-1^ in the control Hoagland’s solution. The greatest rate of decrease in colony counts occurred at 48-h exposure for *P. megasperma* at 0.9 colony per unit of dissolved oxygen reduction (*P* = 0.0015) and at 4-h exposure for *P. pini* at 2.1 (*P* = 0.0002). *Phytophthora nicotianae* and *P. tropicalis* showed an exactly opposite pattern. The colony counts decreased with increasing level of reduction in dissolved oxygen concentration at both 2- and 4-h exposures for *P. nicotianae*, 10-min and 2-h exposures for *P. tropicalis*.

These results indicate that *P. nicotianae* and *P. tropicalis* are more prone than *P. megasperma* and *P. pini* to hypoxia stress in aquatic environments. They help understand the more consistent and greater recoveries of *P. megasperma* and *P. pini* than other major plant pathogens including *P. nicotianae* and *P. tropicalis* in irrigation systems [5,36,37]. Nevertheless, zoospore survival of all four species decreased with increasing intensity of hypoxia. Dissolved oxygen concentration in surface water of irrigation reservoirs can be as low as 0.3 mg L^-1^[13]. This degree of hypoxia is likely to have more pronounced impact on the survival of zoospores in irrigation systems than what observed in this study. The results of present study are critical to understanding the population dynamics of *Phytophthora* species in irrigation reservoirs during hypoxia conditions [36,37].

## Conclusions

In this study we showed for the first time the zoosporic responses to oxygen stress of four economically important species of *Phytophthora* in a simulated aquatic system. Zoospores of these species survived the best in the control solutions at dissolved oxygen concentrations of 5.3 to 5.6 mg L^-1^. Zoospore survival rate decreased with increasing intensity of hyperoxia and hypoxia conditions, depending upon *Phytophthora* species and exposure time. This study also demonstrated that *P. megasperma* had decreasing colony counts with increasing exposure hours from zero to 24 h while the other three species (*P. nicotianae, P. pini* and *P. tropicalis*) had the greatest colony counts at 2 and 4 h during the first 24 h of both elevated and low dissolved oxygen assays. Once again, this study demonstrated that zoospore mortality increases with increasing number of exposure days as did in previous studies [6,7,9]. This natural zoospore decline process was enhanced under hyperoxia and hypoxia conditions. These findings suggest that seasonal and diurnal fluctuations of water quality including dissolved oxygen [13,38] more than likely had contributed to the population decline of *Phytophthora* species along the water path in the same agricultural reservoirs [36,37]. These findings advanced our understanding of aquatic ecology of *Phytophthora* species. They also provided an important basis for pathogen risk avoidance and mitigation by designing better recycling irrigation systems and modifying existing systems to prolong runoff water turnover time.

## Competing interests

The authors declare that they have no competing interests.

## Authors’ contributions

PK designed and performed the experiments. PK and CH analyzed the data and wrote the manuscript together. Both authors read and approved the final manuscript.

## References

[B1] Blackwell E Species of Phytophthora as water moulds Nature 1944 153 496

[B2] Deacon JW Donaldson SP Molecular recognition in the homing responses of zoosporic fungi, with special reference to Pythium and Phytophthora Mycol Res 1993 97 1153 1171 10.1016/S0953-7562(09)81278-1

[B3] Duniway JM Water relation of water molds Ann Rev Phytopathol 1979 17 431 460 10.1146/annurev.py.17.090179.002243

[B4] Erwin DC Ribeiro OK Phytophthora Diseases Worldwide 1996 St Paul, MN, USA: APS Press

[B5] Hong CX Moorman GW Wohanka W Buettner C (eds.): Biology, Detection and Management of Plant Pathogens in Irrigation Water 2014 St. Paul, MN, USA: APS Press

[B6] Kong P Lea-Cox JD Hong CX Effect of electrical conductivity on survival of Phytophthora alni, P. kernoviae and P. ramorum in a simulated aquatic environment Plant Pathol 2012 61 1179 1186 10.1111/j.1365-3059.2012.02614.x 22506539

[B7] Kong P Moorman GW Lea-Cox JD Ross DS Richardson PA Hong CX Zoosporic tolerance to pH stress and its implications for Phytophthora species in aquatic ecosystems Appl Environ Microbiol 2009 75 4307 4314 10.1128/AEM.00119-09 19429548 PMC2704844

[B8] Werres S Wagner S Brand T Kaminski K Seipp D Survival of Phytophthora ramorum in recirculating irrigation water and subsequent infection of Rhododendron and Viburnum Plant Dis 2007 91 1034 1044 10.1094/PDIS-91-8-1034 30780439

[B9] Kong P Lea-Cox JD Moorman GW Hong CX Survival of Phytophthora alni, Phytophthora kernoviae, and Phytophthora ramorum in a simulated aquatic environment at different levels of pH FEMS Microbiol Lett 2012 332 54 60 10.1111/j.1574-6968.2012.02574.x 22506539

[B10] Kong P Carbon dioxide as a potential water disinfestant for Phytophthora disease risk mitigation Plant Dis 2013 97 369 372 10.1094/PDIS-09-12-0844-RE 30722360

[B11] Ahonsi MO Banko TJ Doane SR Demuren AO Copes WE Hong CX Effects of hydrostatic pressure, agitation and CO2 stress on Phytophthora nicotianae zoospore survival Pest Manag Sci 2010 66 696 704 10.1002/ps.1926 20201053

[B12] Jantzen PG Investigating factors that affect dissolved oxygen concentraton in water Amer Biol Teach 1978 40 346 352

[B13] Hong CX Lea-Cox JD Ross DS Moorman GW Richardson PA Ghimire SR Kong P Containment basin water quality fluctuation and implications for crop health management Irrig Sci 2009 29 485 496

[B14] Fenchel T Finlay BJ Ecology and Evolution in Anoxic Worlds 1995 Oxford, UK: Oxford University Press

[B15] Covey RP Effect of oxygen tension on the growth of Phytophthora cactorum Phytopathology 1970 60 358 359 10.1094/Phyto-60-358

[B16] Mitchell DJ Zentmyer GA Effects of oxygen and carbon dioxide tensions on growth of several species of Phytophthora Phytopathology 1971 61 787 791 10.1094/Phyto-61-787

[B17] Klotz LJ Stolzy LH De Wolfe TA Oxygen requirements of three root-rotting fungi in a liquid medium Phytopathology 1963 53 302 305

[B18] Mitchell DJ Zentmyer GA Effects of oxygen and carbon dioxide tensions on sporangium and oospore formation by Phytophthora spp Phytopathology 1971 61 807 811 10.1094/Phyto-61-807

[B19] Dukes PD Apple JL Effect of oxygen and carbon dioxide tension on growth and inoculum potential of Phytophthora parasitica var. nicotianae Phytopathology 1965 55 666 669

[B20] Burgess T McComb J Hardy G Colquhoun I Influence of low oxygen levels in aeroponics chambers on eucalypt roots infected with Phytophthora cinnamomi Plant Dis 1998 82 368 373 10.1094/PDIS.1998.82.4.368 30856882

[B21] Curtis DS Zentmyer GA Effect of oxygen supply on Phytophthora root rot of avocado in nutrient solution Amer J Bot 1949 36 471 474 10.2307/2438081

[B22] Kong P Lea-Cox JD Hong CX, Moorman GW, Wohanka W, Buettner C Water quality dynamics and influences on pathogen mitigation in irrigation reservoirs Biology, Detection and Management of Plant Pathology in Irrigation Water 2014 St Paul, MN, USA: APS Press 333 346

[B23] Ferguson AJ Jeffers SN Detecting multiple species of Phytophthora in container mixes from ornamental crop nurseries Plant Dis 1999 83 1129 1136 10.1094/PDIS.1999.83.12.1129 30841137

[B24] Sherwood RT Hagedorn DJ Effect of oxygen tension on growth of Aphanomyces euteiches Phytopathology 1961 51 492 493

[B25] Brown GE Kennedy BW Effect of oxygen concentration of Pythium seed rot of soybean Phytopathology 1966 56 407 408

[B26] Klotz LJ Stolzy LH DeWolfe TA A method for determining the oxygen requirement of fungi i liquid media Plant Dis Reptr 1962 46 606 608

[B27] Fraedrich SW Tainter FH Effect of dissolved oxygen concentration on the relative susceptibility of shortleaf and loblolly pine root tips to Phytophthora cinnamomi Phytopathology 1989 79 1114 1118 10.1094/Phyto-79-1114

[B28] Curtis DS Chapman HD Zentmyer GA Resume of investigations concerning the oxygen requirements of avocado seedlings including a study of interrelations to nitrite and Phytophthora cinnamomi CA Avocado Soc Yearbook 1949 1949 155 165

[B29] Caldwell J Effects of high partial pressures of oxygen on fungi and bacteria Nature 1965 206 321 323 10.1038/206321a0 5836342

[B30] Gottlieb SF Pakman LM Effect of high oxygen tension on the growth of selected, aerobic, Gram-negative, pathogenic bacteria J Bacteriol 1968 95 1003 1010 5643043 10.1128/jb.95.3.1003-1010.1968 PMC252124

[B31] Charlton ND von Broembsen SL Survival, settling, and lateral dispersal of encysted zoospores of *Phytophthora* spp. in captured irrigation runoff Phytopathology 2000 90 S13

[B32] Pittis JE Colhoun J Isolation and identification of pythiaceous fungi from irrigation water and their pathogenicity to Antirrhinum, tomato and Chamaecyparis lawsoniana Phytopath Z 1984 110 301 318 10.1111/j.1439-0434.1984.tb00070.x

[B33] Stanghellini ME Kim DH Rasmussen SL Rorabaugh PA Control of root rot of peppers caused by Phytophthora capsici with a nonionic surfactant Plant Dis 1996 80 1113 1116 10.1094/PD-80-1113

[B34] Stanghellini ME Rasmussen SL Kim DH Rorabaugh PA Efficacy of nonionic surfactants in the control of zoospore spread of Pythium aphanidermatum in a recirculating hydroponic system Plant Dis 1996 80 422 428 10.1094/PD-80-0422

[B35] Thomson SV Allen RM Occurrence of Phytophthora species and other potential plant pathogens in recycled irrigation water Plant Dis Reptr 1974 58 945 949

[B36] Ghimire SR Richardson PA Kong P Hu JH Lea-Cox JD Ross DS Moorman GW Hong CX Distribution and diversity of Phytophthora species in nursery irrigation reservoir adopting water recycling system during winter months J Phytopathol 2011 159 713 719 10.1111/j.1439-0434.2011.01831.x

[B37] Hong CX Richardson PA Kong P Decline in Phytophthora population with increasing distance from runoff water entrance in a retention pond Phytopathology 2003 93 S36

[B38] Hong CX Richardson PA Ghimire SR Kong P Moorman GW Lea-Cox JD Ross DS Water quality dynamics in irrigation runoff retention basins and its practical implications for plant health management Phytopathology 2008 98 S68

